# A Pilot Study: The Reduction in Fecal Acetate in Obese Patients after Probiotic Administration and Percutaneous Electrical Neurostimulation

**DOI:** 10.3390/nu15051067

**Published:** 2023-02-21

**Authors:** Octavian Parascinet, Sebastián Mas, Tianyu Hang, Carolina Llavero, Óscar Lorenzo, Jaime Ruiz-Tovar

**Affiliations:** 1 Laboratory of Diabetes and Vascular pathology, IIS-Fundación Jiménez Díaz, Medicine Department, Universidad Autónoma, 28040 Madrid, Spain; 2 Spanish Biomedical Research Centre on Diabetes and Associated Metabolic Disorders (CIBERDEM) Network, 28040 Madrid, Spain; 3 Obesity Unit, Clinica Garcilaso, 28010 Madrid, Spain; 4 Department of Medicine, Universidad Alfonso X, 28691 Madrid, Spain

**Keywords:** probiotics, acetate, percutaneous electrical neurostimulation

## Abstract

Previous data suggested that anti-obesity interventions, such as percutaneous electric neurostimulation and probiotics, could reduce body weight and cardiovascular (CV) risk factors by attenuation of microbiota alterations. However, potential mechanisms of action have not been unveiled, and the production of short-chain fatty acids (SCFAs) might be involved in these responses. This pilot study included two groups of class-I obese patients (N = 10, each) who underwent anti-obesity therapy by percutaneous electric neurostimulations (PENS) and a hypocaloric diet (Diet), with/without the administration of the multi-strain probiotic (*Lactobacillus plantarum* LP115, *Lactobacillus acidophilus* LA14, and *Bifidobacterium breve* B3), for ten weeks. Fecal samples were used for SCFA quantification (by HPLC-MS) in relation to microbiota and anthropometric and clinical variables. In these patients, we previously described a further reduction in obesity and CV risk factors (hyperglycemia, dyslipemia) after PENS-Diet+Prob compared to PENS-Diet alone. Herein, we observed that the administration of probiotics decreased fecal acetate concentrations, and this effect may be linked to the enrichment of *Prevotella*, *Bifidobacterium* spp., and *Akkermansia muciniphila*. Additionally, fecal acetate, propionate, and butyrate are associated with each other, suggesting an additional benefit in colonic absorption. In conclusion, probiotics could help anti-obesity interventions by promoting weight loss and reducing CV risk factors. Likely, modification of microbiota and related SCFA, such as acetate, could improve environmental conditions and permeability in the gut.

## 1. Introduction

In fifty years, obesity has increased from 4.8% to 12.9% of the adult population, and from 0.8% to 6.7% in children and adolescents [1,2,3]. Obesity is a complex metabolic pathology caused by several genetic and nongenetic agents, such as environmental factors. It manifests as changes in body appearance but also correlates with glycemic and lipidemic alterations, oxidative stress, chronic inflammation, and increased risk of lethal diseases [3]. In fact, obesity is a major risk factor for type-2 diabetes mellitus (T2DM) and cardiovascular diseases (CVD) [4,5], and the interrelationship between these pathologies may suggest the participation of common mechanisms. Several studies that involved animals and humans have recently demonstrated a striking connection between the development of CVD and an imbalance in the gut microbiota composition (dysbiosis) along with the presence of their derived metabolites [6,7]. Up to 100 trillion symbiotic microbes live in the gut. Healthy microbiota in humans is highly diverse and mainly composed of Firmicutes, Bacteroides, Proteus, Actinomycetes, Fusobacteria, and Verrucomicrobia [8]. Under obesity, bacterial microbiota may suffer alterations in taxonomic diversity and composition, as well as in gut distribution. Notably, a metagenomic study using 16S rRNA gene sequencing revealed microbiome alterations between obese and lean mice [9,10]. Microbiota modifications were linked with two dominant bacterial phyla, Firmicutes and Bacteroidetes. The ratio of Firmicutes/Bacteroidetes has been proposed as a marker for obesity. However, this ratio has been found variable along animal and human studies [11]. Interestingly, these bacteria produce different substrates and metabolites to promote or inhibit the growth of different microorganisms. Additionally, these products can be assimilated into the bloodstream along the intestine leading to different effects on the organism [12,13]. Active elements include short-chain fatty acids (SCFA), vitamins, amino-acids and antioxidant, anti-inflammatory, and analgesic products, as well as potentially harmful agents such as carcinogens and immunotoxins [14,15].

Short-chain fatty acids (SCFAs) are residual metabolites excreted by the gut microbiota after the degradation of dietary fiber and indigestible carbohydrates. Commensal bacteria such as *Bifidobacterium*, *Bacteroides*, *Enterobacter*, *Faecalibacterium*, and *Roseburia* species may be able to ferment these fibers and carbohydrates into SCFA. SCFAs are fatty acids composed of two to six carbons: hexanoic, pentanoic, and more abundantly, acetic, propionic, and butyric acid. They are not only required for the nutritional demands of microorganisms, but also impact host immunity and metabolism as well as regulating local atmosphere conditions and growth of other bacteria [16]. SCFA have been related to beneficial cardiometabolic outcomes in adiposity, glycemia, insulin sensitivity, inflammation, and dyslipemia [16,17,18]. However, after obesity, the levels of fecal and plasma SCFA in clinical studies have been controversially described [19,20,21]. Nevertheless, SCFA-producing microbiota might account as a promising target to control metabolic alterations under obesity. Some multi-strain probiotic made of *Lactobacillus* and/or *Bifidobacterium* have enhanced obesity and associated CV risk factors in clinical trials and animal models [22,23,24,25,26,27]. Other approaches like the percutaneous electro-neurostimulation of the T6 dermatome (PENS) led to weight loss by production of a somato-autonomic reflex that slow stomach emptying and induce early satiety [28]. This intervention can increase patient adherence to diet by regulation of growth hormones, ghrelin, and IGF-1 [28,29]. In fact, in a previous report [30], we described that addition of probiotics to PENS under hypocaloric diet further improved weight loss and the glycemic and lipid profile in class-I obese patients, in parallel to an enrichment of specific bacteria. However, potential mechanisms of these anti-obesity interventions have not been elucidated. Herein, our aim was to seek for a metabolic link between those microbiota alterations and the beneficial outcomes produced by the probiotic administration.

## 2. Materials and Methods

### 2.1. The Pilot Study

As described in Lorenzo et al. [26], this pilot study (NCT03872245) was performed in the Obesity Unit of the Garcilaso Clinic in Madrid (Spain), including two groups of class-I obese patients (N = 10, each) with a female/male ratio of 2.33 in both cases, who underwent anti-obesity therapy by percutaneous electric neurostimulations (PENS) and hypocaloric diet (Diet), with/without administration of the multi-strain probiotic (Adomelle^®^; Bromatech, Milan, Italy). Exclusion criteria were (a) untreated endocrine diseases causing obesity, (b) previous treatment with hormones, prebiotics, probiotics, or with nutritional supplements, (c) diagnosis of previous CVD or cancer, or (d) portable electrical devices.

### 2.2. PENS, Hypocaloric Diet, and Probiotic Administration

Patients who previously were unsuccessfully treated only with the hypocaloric diet were randomly assigned to the PENS-Diet or PENS-Diet+Prob for ten consecutive weeks. The PENS of dermatome T6 was performed by using the Urgent PC 200 Neuromodulation System® (Uroplasty, Minnetonka, MN, USA), as previously described [30]. Patients were placed in a supine position and PENS was delivered by a needle electrode inserted in the left upper quadrant along the medio-clavicular line at two centimeters below the ribcage and at 0.5–1 cm of depth. The PENS was undertaken at a frequency of 20 Hz at the highest amplify (0–20 mA) without causing any pain. The participants underwent one 30-min session every week for ten consecutive weeks. In addition, a 1200 Kcal/day diet was uni-formly prescribed during PENS interventions in both groups of patients, as previously described [30]. The diet followed a Mediterranean style (carbohydrates 51%, proteins 23% and fat 26%) with a high intake of fruit and vegetables, a moderate intake of meats, and olive oil as the main source of fat. A record of food intake was applied along the study. Also, all patients followed an exercise activity of 1h/day brisk walking. The multi-strain probiotic consisted of a mixture of *Lactobacillus plantarum* LP115 (<1 × 10^9^ colony forming units, CFU), *Lactobacillus acidophilus* LA14 (1 × 10^9^ CFU), and *Bifidobacterium breve* B3 (<1 × 10^9^ CFU). It was given (2 tablets/day) with water after meals, without altering the amount of food intake. Additionally, all patients followed an exercise activity of 1 h/day brisk walking. The work was carried out in accordance with The Code of Ethics of the World Medical Association (Declaration of Helsinki). The Ethical Committee of Clinical Research (Medicine, Esthetic, and Longevity Foundation) approved this investigation (ref.: Garcilas-19-3; Feb 2019). 

### 2.3. Clinical and Microbiota Variables

Clinical variables such as BMI (kg/m^2^), weight loss (WL), the percentage of total weight lost (%TWL), the percentage of excess BMI lost (%EBMIL), systolic (SBP) and diastolic (DBP) blood pressure, fasting glucose, glycated hemoglobin (HbA1c), and the lipid profile [triglycerides (TG), total cholesterol, LDL-cholesterol (LDLc), HDL-cholesterol (HDLc)] were measured at the Clinical Analytical Department of the Hospital Fundación Jiménez Díaz. Additionally, fecal samples were isolated and frozen (−80 °C) before and after the PENS-Diet or PENS-Diet+Prob treatments to analyze the intestinal microbiota [30] and the composition of short-chain fatty acids (SCFAs). 

### 2.4. Fecal SCFA Quantification

Fecal samples were thawed and derivatized with 3-nitrophenylhydrazine (3-NPH), as described by Han et al. [31]. The derivatization process started by mixing 50 μL of fecal matter with 50 μL of AcN (50%) in deionized water. The mixture was then centrifuged for 10 min at 5000 g under 4 °C. Forty μL of the supernatant was mixed with 20 μL of 200 mM 3-NPH, 20 μL of 120 mM 1-Ethyl-3-(3-dimethylaminopropyl) carbodiimide (EDC), and 6% pyridine (Sigma-Aldrich, Burlington, USA). They were incubated for 30 min at 40 °C. Finally, 920 μL of AcN (10%) in deionized water was added into each tube and samples were frozen at −30 °C until HPLC-MS analysis. The standard samples of SCFA (acetic, propionic, and butyric acid) were prepared in a 50% AcN:H_2_O solution and derivatization was carried out in the same manner as fecal samples. 

The analysis for acetate, propionate, and butyrate was performed by LC-MS/MS at the mass spectrometry facility of Complutense University (Madrid). The quantitative analysis by MRM used an LC-ESI-QQQ 8030 Shimadzu mass spectrometer and a Phenomenex Gemini 5 μm C18 110 A 150 × 2 mm column (Agilent, Santa Clara, USA). A phase gradient was applied to a 20 μL injection volume: phase A (H_2_O + 0.01% formic acid) and phase B (AcN + 0.01% formic acid). The flow rate of the mobile phase was stabilized at 0.6 mL/min, and the total elution time of the compounds was set at 11 min. Firstly, phase B was applied at 20% for 2 min, after which phase B was set up to 40% for 5 additional minutes. Then, phase B was raised from 40% to 100% (from minutes 8 to 11) and later, it returned to the initial conditions (Supplementary Table S1A). Mass spectra of the parental and fragmented ions were used for quality and quantity determination of SCFAs (Supplementary Table S1B).

### 2.5. Statistical Analysis

The statistical analyses were performed by R 4.1.1 software. The Shapiro–Wilk test was used to analyze the normality of variables. Then, non-parametric tests were used for all variables. Within each group (PENS-Diet or PENS-Diet+Prob), the Wilcoxon signed-rank test was used to compare median values of metabolite concentrations before and after treatment. The Mann–Whitney test was performed to compare the differential values of each metabolite between the PENS-Diet and PENS-Diet+Prob treatments. Spearman’s correlation was used to analyze the relationship between SCFA concentration and clinical and microbiota variables. Finally, a quantile regression analysis was performed for variables showing greater association in Spearman’s correlation.

## 3. Results

### 3.1. Probiotics Administration Further Reduced Obesity and CV Risk Factors

The characterization of this pilot study was previously published by Lorenzo et al. [30]. Briefly, at baseline, there were no significant differences in BMI, age, and sex between the PENS-Diet and PENS-Diet+Prob groups. After treatments, PENS-Diet induced a significant reduction in body weight, systolic and diastolic blood pressure, fasting glucose, plasma triglycerides, and total cholesterol (Table 1a). However, PENS-Diet+Prob triggered a further improvement in weight loss, %TWL, and %EBMIL, and a reduction in plasma HbA1c and triglycerides, in addition to elevated HDLc levels (Table 1a). Additionally, there was a significant association between probiotic administration and the differences between these factors (not shown). Thus, the addition of probiotics to the PENS-Diet promoted a higher enhancement against obesity and cardiovascular risk factors [30]. However, the probiotics-derived mechanisms of action are not fully known, and modification of microbiota and their metabolites could be involved.

### 3.2. Probiotics Induced Microbiota Alterations and Decreased Fecal Acetate

**Figure 1:**
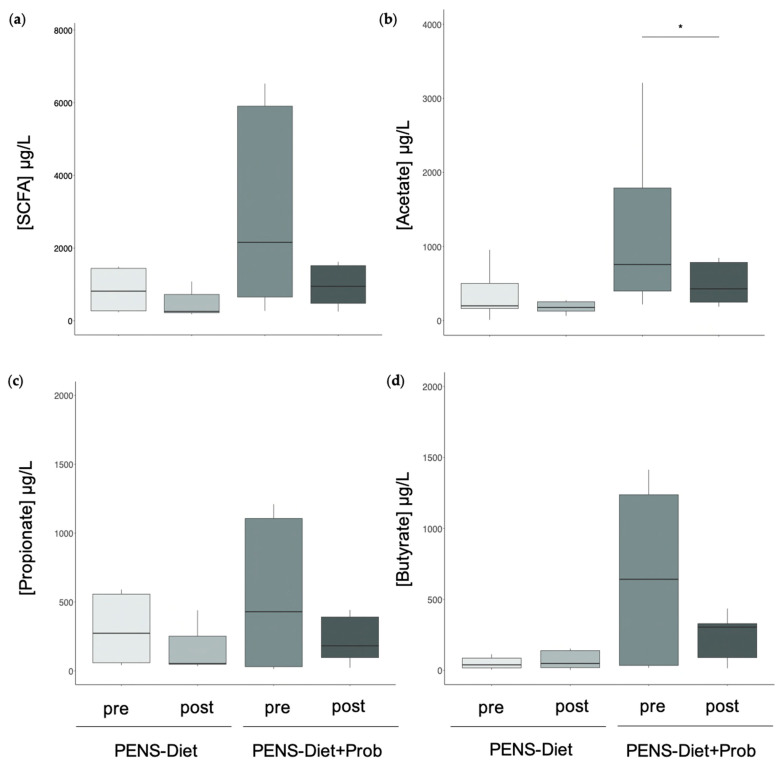
Fecal levels of short-chain fatty acids after PENS-Diet and PENS-Diet+Prob. (**a**) Total levels of main SCFAs, including acetate, propionate, and butyrate, were quantified by HPLC-MS after ten weeks of PENS-Diet and PENS-Diet+Prob. Individual (**b**) acetate, (**c**) propionate, and (**d**) butyrate concentrations are also shown. * *p* < 0.05 post-treatment vs. pre-treatment.

**Figure 2:**
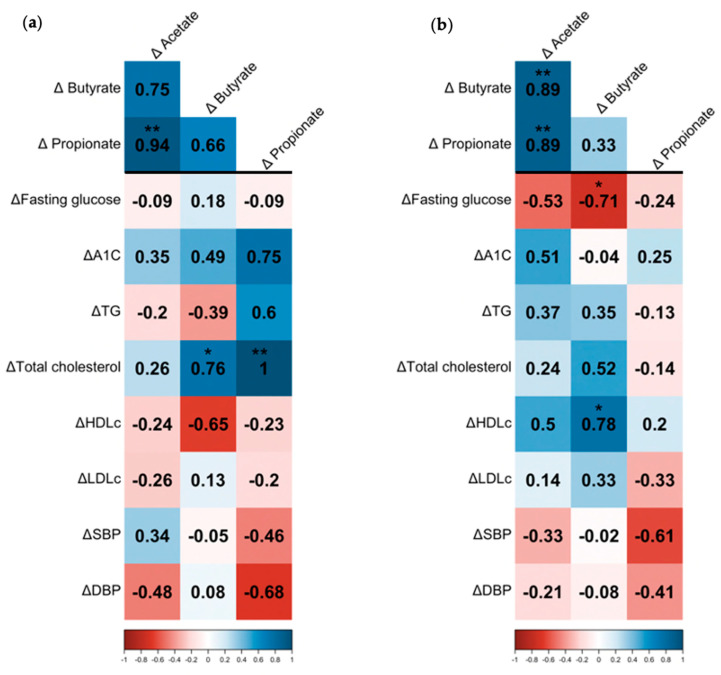
Correlation between SCFAs and clinical outcomes in treated-obese patients. Spearman’s matrix correlations between differential SCFA levels and changes in SCFAs and clinical (fasting glucose, HbA1c, TG, total cholesterol, HDLc, LDLc, SBP, and DBP) parameters in (**a**) PENS-Diet and (**b**) PENS-Diet+Prob. The color scale indicated the lower (−1; red) and the higher (1; blue) Rho values. * *p* < 0.05 and ** *p* < 0.01.

**Figure 3:**
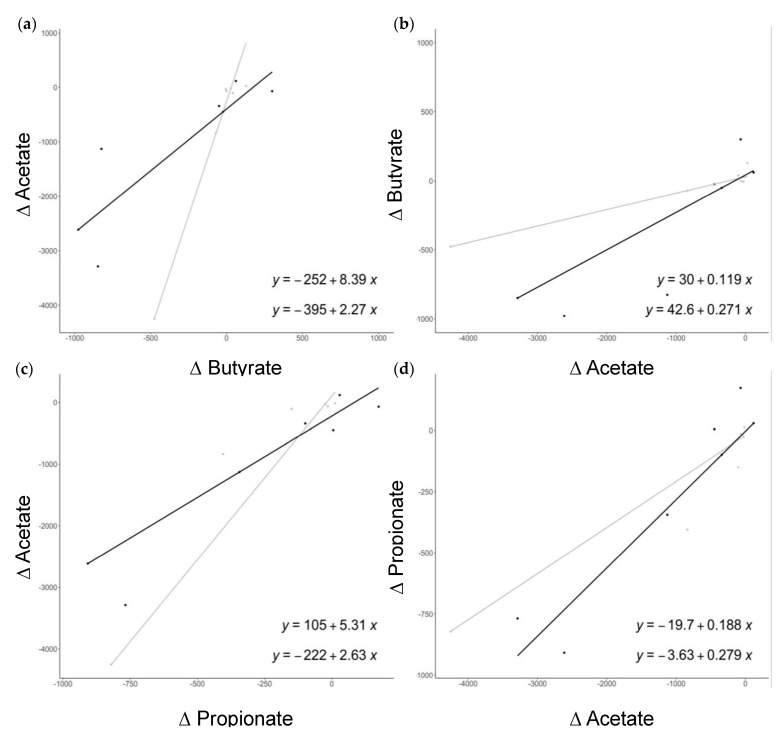
Associations among SCFAs in obese patients after PENS-Diet and PENS-Diet+Prob. The significant quantile regression models for differences (pre- and post-treatment) in (**a**) acetate vs. butyrate, (**b**) butyrate vs. acetate, (**c**) acetate vs. propionate, and (**d**) propionate vs. acetate. In the grey line, the PENS-Diet model. In the black line, PENS-Diet+Prob. The model equations for PENS-Diet (top) and PENS-Diet+Prob (bottom) are also shown.

As described [30], the PENS-Diet+Prob intervention significantly reduced the Firmicutes/Bacteroidetes ratio and enriched *Prevotella* spp., *A. muciniphila,* and *Bifidobacterium* spp. compared to PENS-Diet (Table 1b). Interestingly, *Bifidobacterium* spp. and *A. muciniphila* have been associated with improvements in gut dysbiosis, cardiometabolic markers, and insulin resistance by the regulation of fecal and plasma SCFA [20,32,33]. Thus, we next quantified the levels of the most abundant fecal SCFA in our patients. In PENS-Diet, we detected non-significant variations of SCFA between before and after treatment, whereas in PENS-Diet+Prob subjects, acetate was significantly lessened (−54%, *p* = 0.023) and butyrate and propionate exhibited a reduction trend (Figure 1). Additionally, both the PENS-Diet and PENS-Diet+Prob groups exhibited significant positive correlations between changes in SCFAs. In PENS-Diet, differential acetate significantly correlated with propionate (Rho = 0.94, *p* < 0.01) (Figure 2a), while in PENS-Diet+Prob, differential acetate was also significantly linked to butyrate (Rho = 0.89, *p* < 0.01) and propionate (Rho = 0.89, *p* < 0.01) (Figure 2b). Thus, fecal SCFAs, particularly acetate and butyrate, may be decreased by gut microbiota after probiotics administration. In this regard, by univariate quantile regression, we found that in PENS-Diet, differential acetate and butyrate were significantly associated with each other (β = 8.39, *p* < 0.01, and β = 0.119, *p* < 0.01) (Figure 3a,b), and acetate was associated with propionate (β = 5.31, *p* = 0.059) (Figure 3c). In PENS-Diet+Prob, differential acetate was associated with butyrate (β = 2.27, *p* = 0.078) (Figure 3a,b) and was propionate in both ways (β = 2.63, *p* < 0.01 and β = 0.28, *p* < 0.01) (Figure 3c,d).

### 3.3. Association between SCFAs, Bacterial Microbiota, and Cardiovascular Risk Factors 

**Figure 4:**
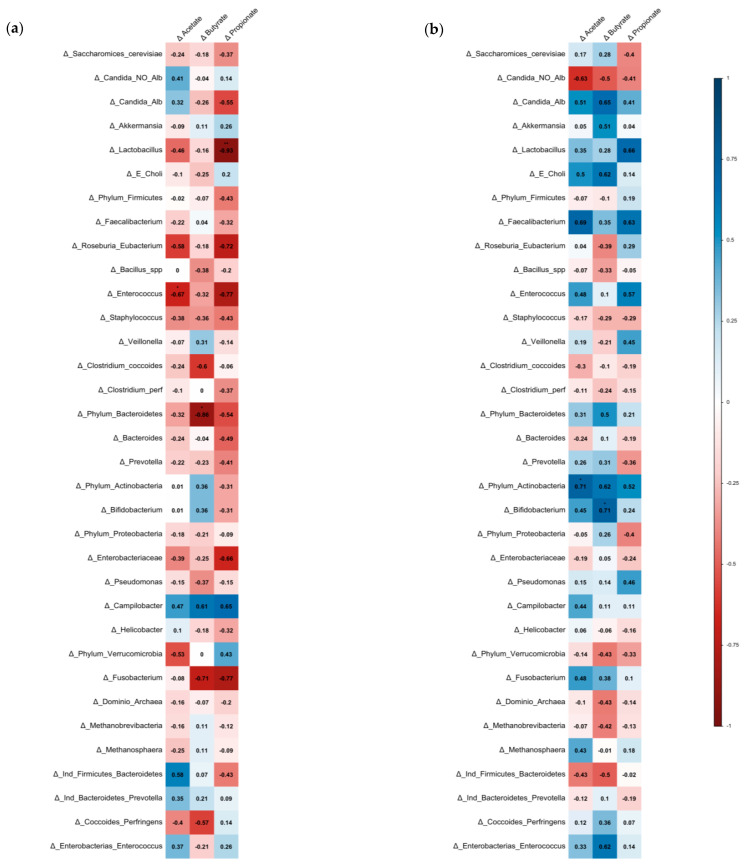
Correlation between SCFAs and changes of microbiota in treated-obese patients. Spearman’s matrix correlations between differential SCFA levels and variations in abundance of specific gut bacteria after (**a**) PENS-Diet or (**b**) PENS-Diet+Prob. The color scale indicated the lower (−1; red) and the higher (1; blue) Rho values. * *p* < 0.05 and ** *p* < 0.01.

The reduction in fecal SCFAs might be associated with the enrichment of specific bacteria and with the improvement of clinical outcomes in obese patients. In PENS-Diet subjects, a negative correlation between differential acetate and *Enterococcus* (Rho = −0.67, *p* = 0.035) was noted. Additionally, propionate was inversely correlated with *Lactobacillus* (Rho = −0.93, *p* = 0.008) and butyrate with Bacteroidetes (Rho = −0.86, *p* = 0.014) (Figure 4a). Interestingly, butyrate and propionate concentrations directly correlated with total cholesterol (Rho = 0.76, *p* = 0.049 and Rho = 1, *p* < 0.01, respectively) (Figure 2a). On the other hand, in PENS-Diet+Prob, reductions in acetate could be associated with Actinobacteria decrease (Rho = 0.71, *p* = 0.047), while butyrate levels might be positively linked to *Bifidobacterium* spp. (Rho = 0.71, *p* = 0.047) (Figure 4b). However, fecal butyrate might be inversely correlated with fasting glucose (Rho = −0.71, *p* = 0.047) and directly with HDLc (Rho = 0.78, *p* = 0.023) (Figure 2b). 

## 4. Discussion

In this pilot study, an anti-obesity intervention by PENS and a hypocaloric diet for ten weeks induced weight loss and improvement of blood pressure, glycemia, and hyperlipidemia in class-I obese patients. Importantly, the concomitant administration of probiotics (*L. plantarum*, *L. acidophilus*, and *B. breve* B3) led to further amelioration of these parameters. These probiotics enhanced the growth of *Prevotella* spp., *Bifidobacterium* spp., and *A. muciniphila*, and reduced the Firmicutes/Bacteroidetes ratio. As a potential consequence, the SCFA acetate decreased in fecal samples and this effect could be linked with clinical outcomes.

SCFA can induce anorexigenic and insulinotropic peptides (i.e., leptin, PYY, GLP-1) and stimulate anti-inflammatory responses [16,17]. In obese mice, exogenous administration of butyrate reduced hepatic steatosis and inflammation, improving the gut barrier integrity [34]. Both propionate and butyrate increased plasma incretins and insulin sensitivity [17], and acetate enhanced cardiac hypertrophy, insulin sensitivity, and oxidative stress, and elevated plasma HDLc levels [35]. Also in these mice, amelioration of obesity was associated to the probiotics stimulated fecal production of SCFA [25,26]. However, after obesity, the levels of fecal SCFA (acetate, propionate, and butyrate) have been controversially described. A reduction of SCFA has been mostly observed in obese rodents [25,26], but in human obesity, variable concentrations of SCFA have been unveiled [19,20,21]. The concentration of fecal SCFA is inherently derived from their production and absorption rates. Most of SCFA absorption is in proximal colon and thus, caecal SCFA levels are directly correlated with their concentrations [36]. In contrast, an inverse link between fecal SCFA (i.e., acetate) and their absorption rate was previously reported [37]. Likely, gut barrier can be disturbed in obesity by alterations in microbiota, mucus, immune system, and environmental conditions (pH, water, ions) [38], and thus, SCFA permissibility and their potential benefits could be diminished. In obese subjects, the higher presence of stool SCFA were associated with reductions in *A. muciniphila* and *Bacteroides*, and increased blood pressure, proinflammatory markers, and the lipid/glycemic profiles [21]. *A. muciniphila* has been described as a mucin-degrading bacteria with protective roles on intestinal gut barrier [39,40]. 

In this regard, reconstitution of unbalanced microbiota may be achieved by enrich-ment with specific bacteria from probiotics. Multi-strain formula of probiotics has elicited favorable activities against metabolic and cardiovascular diseases. They improved body weight, insulin resistance, GLP-1 release, and hyperlipidemia [8]. Previous reports have tested the combination of both *Lactobacillus* and *Bifidobacterium* probiotics in diet-induced obese mice [41]. Remarkedly, this combination led to higher weight loss and hypoli-pidemic effects than probiotics alone. Thus, multi-strain probiotics may induce faster growing and stabilization of their bacteria and trigger synergetic actions on host intestine by metabolites production, which could lead to attenuation of metabolic and cardiovas-cular risk factors. In this sense, a multi-strain probiotic made of *Lactobacillus* and *Entero-coccus* produced higher concentrations of SCFA (i.e., acetate and butyrate) than each bacte-rium alone [42]. Probiotics might also enhance other SCFA-producing bacteria and in-crease SCFA permeability at the intestine [43,44]. SCFA could promote gut barrier repair by triggering other bacteria and enterocytes and colonocytes growing [16,45]. The close correlation between acetate and butyrate levels also suggests their positive action on intes-tinal permeability. In this line, *Bifidobacterium* spp. and *A. muciniphila* can generate acetate and promote butyrate synthesis by other bacteria [46,47]. In our study, PENS-Diet+Prob, but not PENS-Diet alone, enriched *Prevotella* spp., *Bifidobacterium* spp., and *A. muciniphila*, which could encourage gut barrier integrity by balancing microbiota and releasing acetate. Then, acetate and other SCFA might be better assimilated to promote anorexigenic, insulinotropic, and anti-inflammatory responses, helping on the reduction in body weight, glycemia, and hyperlipidemia [34]. Also, acetate-consuming bacteria with favorable actions would have obtained nutrients to grow and regain gut eubiosis. Interestingly, we found only significant reductions for fecal acetate and tendencies to decrease for propionate and butyrate, after probiotics. Likely, longer treatments of these probiotics (or others) might have influenced also on more SCFA. In this sense, administration of *Lactobacillus rhamnosus* for 20 weeks in obese women provoked a decrease in both fecal acetate and butyrate [48]. Moreover, acetate may be more sensitive to obesity, diet modifications or probiotics than other SCFA. De la cuesta-Zuluaga et al described a greater increase of acetate than that of propionate and butyrate in overweight/class-I obese individuals, compared to their lean counterparts [21], and higher degrees of obesity have been associated with elevation of several fecal SCFA (acetate, propionate, and butyrate) [19]. Altogether, this multi-strain probiotics may reduce obesity and CVD risk factors at least in part by increasing *Bifidobacterium* spp. and *A. muciniphila* and derivate SCFA like acetate.

### Limitations of the Study

Although this is a pilot study, an obvious limitation is the reduced sampling size which can influence statistical power. Additionally, a group of subjects who follow only a diet regime, PENS intervention, or probiotic intake could offer comparative data about microbiota distribution and metabolite release. Since multiple factors (presence of comorbidities, habits, etc.) could influence probiotics and SCFA actions, our data should be taken with care. Finally, a direct comparison of SCFA levels in plasma and fecal samples and the analysis of gut tissue could quantify alterations in SCFA absorption under obesity and after treatments. All these variables will be considered in a future study.

## 5. Conclusions

Administration of probiotics could be useful at least for coadjutant therapy for ameliorating body weight and CVD risk factors under obesity. Probiotics may enrich specific bacteria and change microbiota composition and distribution along the intestine. A mix of probiotics *Lactobacillus plantarum*, *Lactobacillus acidophilus*, and *Bifidobacterium breve* B3 induced the growth of *Prevotella* spp., *Bifidobacterium* spp., and *A. muciniphila.* Interestingly, some of these bacteria can produce metabolites, such as acetate, with potential cardioprotective actions (hypolipidemic, insulinotropic, anti-inflammatory). In turn, acetate might enhance the gut environment and permeability to selective nutrients and metabolites, and thus, it could favor their assimilation to the systemic circulation. More clinical assays are required to investigate the gut absorption rates and potential cardioprotective actions of SCFA under obesity, with and without probiotic administration.

## Figures and Tables

**Table 1:** Clinical and microbiota outcomes in obese patients after PENS-Diet and PENS-Diet+Prob interventions.

(**a**)		
	**PENS-Diet**	**PENS-Diet+Prob**
WL (kg)	11.1 ± 4.4 **	16.2 ± 4.6 ** #
%TWL	12.9 ± 4.5	17.5 ± 3.5 #
%EBMIL	57.0 ± 12.3	84.2 ± 29.5 #
Δ SBP (mmHg)	−12.5 (22.5) *	−10.0 (12.5) *
Δ DBP (mmHg)	−10.0 (10.0) *	−2.5 (10.0) *
Δ Fasting glucose (mg/dL)	−7.0 (11.0) **	−13.0 (16.5) *
Δ HbA1c (%)	−0.05 ± 0.4	−0.46 ± 0.4 * #
Δ TG (mg/dL)	−8.5 (26.0) **	−47.0 (63.75) ** ##
Δ Total cholesterol (mg/dL)	−9.0 ± 7.4 **	−18.5 ± 33.3
Δ LDLc (mg/dL)	0.5 (42.75)	−18.0 (25.5)
Δ HDLc (mg/dL)	0.05 (6.8)	10.5 (12) ##
(**b**)		
	**PENS-Diet**	**PENS-Diet+Prob**
*Prevotella* spp.	−0.15 (1.3)	1.05 (2.6) #
*Bifidobacterium* spp.	0.08 ± 2.2	1.70 ± 1.4 ** #
*Akkermansia muciniphila*	0.20 (0.4)	1.05 (2.1) * ##
Firmicutes/Bacteroidetes	−0.04 ± 0.2	−0.36 ± 0.4 * #

(**a**) The differences between pre- and post-treatment in weight loss (WL), percentage of total weight loss (%TWL), percentage of the excess of body mass index loss (%EBMIL), systolic blood pressure (SBP), diastolic blood pressure (DBP), and glycemic and lipid parameters are shown for PENS-Diet and PENS-Diet+Prob patients. (**b**) Microbiota differences between pre- and post-treatments in both groups of patients. Values are shown as median (IQR) or mean ± SD. * *p* < 0.05 and ** *p* < 0.01 post-treatment vs. pre-treatment. # *p* < 0.05 and ## *p* < 0.01 PENS-Diet+Prob vs. PENS-Diet. Glycated hemoglobin A1c, HbA1c; triglycerides, TG; HDL—cholesterol, HDLc; LDL—cholesterol, LDLc; systolic blood pressure, SBP; diastolic blood pressure, DBP.

## Supplementary Materials

The following supporting information can be downloaded at https://www.mdpi.com/article/10.3390/nu15051067/s1. Supplementary Table S1. (A) Mobile phase gradient method for SCFA analysis and (B) transition pairs of SCFA by HPLC-MS analysis.

## References

[1] Chooi Y.C., Ding C., Magkos F. (2019). The epidemiology of obesity. Metabolism.

[2] Blüher M. (2019). Obesity: Global epidemiology and pathogenesis. Nat. Rev. Endocrinol..

[3] Arroyo-Johnson C., Mincey K.D. (2016). Obesity Epidemiology Worldwide. Gastroenterol. Clin. N. Am..

[4] Kahn S.E., Hull R.L., Utzschneider K.M. (2006). Mechanisms linking obesity to insulin resistance and type 2 diabetes. Nature.

[5] Ortega F.B., Lavie C.J., Blair S.N. (2016). Obesity and cardiovascular disease. Circ. Res..

[6] Aron-Wisnewsky J., Warmbrunn M.V., Nieuwdorp M., Clément K. (2021). Metabolism and Metabolic Disorders and the Microbiome: The Intestinal Microbiota Associated With Obesity, Lipid Metabolism, and Metabolic Health—Pathophysiology and Therapeutic Strategies. Gastroenterology.

[7] Jin M., Qian Z., Yin J., Xu W., Zhou X. (2019). The role of intestinal microbiota in cardiovascular disease. J. Cell. Mol. Med..

[8] Abenavoli L., Scarpellini E., Colica C., Boccuto L., Salehi B., Sharifi-Rad J., Aiello V., Romano B., De Lorenzo A., Izzo A.A. (2019). Gut Microbiota and Obesity: A Role for Probiotics. Nutrients.

[9] Liu R., Hong J., Xu X., Feng Q., Zhang D., Gu Y., Shi J., Zhao S., Liu W., Wang X. (2017). Gut microbiome and serum metabolome alterations in obesity and after weight-loss intervention. Nat. Med..

[10] Turnbaugh P.J., Ley R.E., Mahowald M.A., Magrini V., Mardis E.R., Gordon J.I. (2006). An Obesity-Associated Gut Microbiome with Increased Capacity for Energy Harvest. Nature.

[11] Magne F., Gotteland M., Gauthier L., Zazueta A., Pesoa S., Navarrete P., Balamurugan R. (2020). The firmicutes/bacteroidetes ratio: A relevant marker of gut dysbiosis in obese patients?. Nutrients.

[12] Cho C.E., Taesuwan S., Malysheva O.V., Bender E., Tulchinsky N.F., Yan J., Sutter J.L., Caudill M.A. (2016). Trimethylamine-*N*-oxide (TMAO) response to animal source foods varies among healthy young men and is influenced by their gut microbiota composition: A randomized controlled trial. Mol. Nutr. Food Res..

[13] Kasahara K., Krautkramer K.A., Org E., Romano K.A., Kerby R.L., Vivas E.I., Mehrabian M., Denu J.M., Bäckhed F., Lusis A.J. (2018). Interactions between Roseburia intestinalis and diet modulate atherogenesis in a murine model. Nat. Microbiol..

[14] Croci S., D’apolito L.I., Gasperi V., Catani M.V., Savini I. (2021). Dietary strategies for management of metabolic syndrome: Role of gut microbiota metabolites. Nutrients.

[15] Coker O.O., Liu C., Wu WK K., Wong S.H., Jia W., Sung J.J., Yu J. (2022). Altered gut metabolites and microbiota interactions are implicated in colorectal carcinogenesis and can be non-invasive diagnostic biomarkers. Microbiome.

[16] Tan J., McKenzie C., Potamitis M., Thorburn A.N., Mackay C.R., Macia L. (2014). The Role of Short-Chain Fatty Acids in Health and Disease. Adv. Immunol..

[17] Lin H.V., Frassetto A., Kowalik E.J., Nawrocki A.R., Lu M.M., Kosinski J.R., Hubert J.A., Szeto D., Yao X., Forrest G. (2012). Butyrate and propionate protect against diet-induced obesity and regulate gut hormones via free fatty acid receptor 3-independent mechanisms. PLoS ONE.

[18] Ang Z., Ding J.L. (2016). GPR41 and GPR43 in obesity and inflammation—Protective or causative?. Front. Immunol..

[19] Kim K.N., Yao Y., Ju S.Y. (2019). Short chain fatty acids and fecal microbiota abundance in humans with obesity: A systematic review and meta-analysis. Nutrients.

[20] Nagata S., Chiba Y., Wang C., Yamashiro Y. (2017). The effects of the *Lactobacillus casei* strain on obesity in children: A pilot study. Benef. Microbes.

[21] de la Cuesta-Zuluaga J., Mueller N.T., Álvarez-Quintero R., Velásquez-Mejía E.P., Sierra J.A., Corrales-Agudelo V., Carmona J.A., Abad J.M., Escobar J.S. (2019). Higher fecal short-chain fatty acid levels are associated with gut microbiome dysbiosis, obesity, hypertension and cardiometabolic disease risk factors. Nutrients.

[22] Li Z., Yang S., Lin H., Huang J., Watkins P.A., Moser A.B., DeSimone C., Song X., Diehl A.M. (2003). Probiotics and antibodies to TNF inhibit inflammatory activity and improve nonalcoholic fatty liver disease. Hepatology.

[23] Yadav H., Lee J.-H., Lloyd J., Walter P., Rane S.G. (2013). Beneficial Metabolic Effects of a Probiotic via Butyrate-induced GLP-1 Hormone Secretion. J. Biol. Chem..

[24] López-Moreno A., Suárez A., Avanzi C., Monteoliva-Sánchez M., Aguilera M. (2020). Probiotic Strains and Intervention Total Doses for Modulating Obesity-Related Microbiota Dysbiosis: A Systematic Review and Meta-Analysis. Nutrients.

[25] Ji Y., Park S., Chung Y., Kim B., Park H., Huang E., Jeong D., Jung H.-Y., Kim B., Hyun C.-K. (2019). Amelioration of obesity-related biomarkers by Lactobacillus sakei CJLS03 in a high-fat diet-induced obese murine model. Sci. Rep..

[26] Horiuchi H., Kamikado K., Aoki R., Suganuma N., Nishijima T., Nakatani A., Kimura I. (2020). Bifidobacterium animalis subsp. lactis GCL2505 modulates host energy metabolism via the short-chain fatty acid receptor GPR43. Sci. Rep..

[27] Wang J., Ji H., Wang S., Liu H., Zhang W., Zhang D., Wang Y. (2018). Probiotic Lactobacillus plantarum Promotes Intestinal Barrier Function by Strengthening the Epithelium and Modulating Gut Microbiota. Front. Microbiol..

[28] Ruiz-Tovar J., Llavero C., Smith W. (2017). Percutaneous Electrical Neurostimulation of Dermatome T6 for Short-term Weight Loss in Overweight and Obese Patients: Effect on Ghrelin Levels, Glucose, Lipid, and Hormonal Profile. Surg. Laparosc. Endosc. Percutaneous Tech..

[29] Giner-Bernal L., Ruiz-Tovar J., Violeta J., Mercader M., Miralles J., Calpena R., Arroyo A. (2020). Plasma ghrelin levels after percutaneous electrical nerve stimulation of dermatome T6 for the treatment of obesity. Endocrinol. Diabetes Y Nutr..

[30] Lorenzo O., Crespo-Yanguas M., Hang T., Lumpuy-Castillo J., Hernández A.M., Llavero C., García-Alonso M., Ruiz-Tovar C. (2020). Addition of probiotics to anti-obesity therapy by percutaneous electrical stimulation of dermatome T6. A pilot study. Int. J. Environ. Res. Public Health.

[31] Han J., Lin K., Sequeira C., Borchers C.H. (2015). An isotope-labeled chemical derivatization method for the quantitation of short-chain fatty acids in human feces by liquid chromatography–tandem mass spectrometry. Anal. Chim. Acta.

[32] Amabebe E., Robert F.O., Agbalalah T., Orubu E.S.F. (2020). Microbial dysbiosis-induced obesity: Role of gut microbiota in homoeostasis of energy metabolism. Br. J. Nutr..

[33] Rodrigues V.F., Elias-Oliveira J., Pereira S., Pereira J.A., Barbosa S.C., Machado M.S.G., Carlos D. (2022). Akkermansia muciniphila and Gut Immune System: A Good Friendship That Attenuates Inflammatory Bowel Disease, Obesity, and Diabetes. Front. Immunol..

[34] Fang W., Xue H., Chen X., Chen K., Ling W. (2019). Supplementation with Sodium Butyrate Modulates the Composition of the Gut Microbiota and Ameliorates High-Fat Diet-Induced Obesity in Mice. J. Nutr..

[35] Olaniyi K.S., Amusa O.A., Areola E.D., Olatunji L.A. (2020). Suppression of HDAC by sodium acetate rectifies cardiac metabolic disturbance in streptozotocin–nicotinamide-induced diabetic rats. Exp. Biol. Med..

[36] Jakobsdottir G., Jädert C., Holm L., Nyman M.E. (2013). Propionic and butyric acids, formed in the caecum of rats fed highly fermentable dietary fibre, are reflected in portal and aortic serum. Br. J. Nutr..

[37] Vogt J.A., Wolever T.M.S. (2003). Fecal Acetate Is Inversely Related to Acetate Absorption from the Human Rectum and Distal Colon. J. Nutr..

[38] Lee C.J., Sears C.L., Maruthur N. (2020). Gut microbiome and its role in obesity and insulin resistance. Ann. N. Y. Acad. Sci..

[39] de La Cuesta-Zuluaga J., Mueller N.T., Corrales-Agudelo V., Velásquez-Mejía E.P., Carmona J.A., Abad J.M., Escobar J.S. (2017). Metformin is associated with higher relative abundance of mucin-degrading akkermansia muciniphila and several short-chain fatty acid-producing microbiota in the gut. Diabetes Care.

[40] Everard A., Belzer C., Geurts L., Ouwerkerk J.P., Druart C., Bindels L.B., Guiot Y., Derrien M., Muccioli G.G., Delzenne N.M. (2013). Cross-talk between *Akkermansia muciniphila* and intestinal epithelium controls diet-induced obesity. Proc. Natl. Acad. Sci. USA.

[41] Bubnov R.V., Babenko L.P., Lazarenko L.M., Mokrozub V.V., Demchenko O.A., Nechypurenko O.V., Spivak M.Y. (2017). Comparative study of probiotic effects of Lactobacillus and Bifidobacteria strains on cholesterol levels, liver morphology and the gut microbiota in obese mice. EPMA J..

[42] Nagpal R., Wang S., Ahmadi S., Hayes J., Gagliano J., Subashchandrabose S., Kitzman D.W., Becton T., Read R., Yadav H. (2018). Human-origin probiotic cocktail increases short-chain fatty acid production via modulation of mice and human gut microbiome. Sci. Rep..

[43] Schwiertz A., Taras D., Schäfer K., Beijer S., Bos N.A., Donus C., Hardt P.D. (2010). Microbiota and SCFA in Lean and Overweight Healthy Subjects. Obesity.

[44] Kong C., Gao R., Yan X., Huang L., Qin H. (2019). Probiotics improve gut microbiota dysbiosis in obese mice fed a high-fat or high-sucrose diet. Nutrition.

[45] Zou J., Chassaing B., Singh V., Pellizzon M., Ricci M., Fythe M.D., Kumar M.V., Gewirtz A.T. (2018). Fiber-Mediated Nourishment of Gut Microbiota Protects against Diet-Induced Obesity by Restoring IL-22-Mediated Colonic Health. Cell Host Microbe.

[46] Rios-Covian D., Gueimonde M., Duncan S.H., Flint H.J., de Los Reyes-Gavilan C.G. (2015). Enhanced butyrate formation by cross-feeding between Faecalibacterium prausnitzii and Bifidobacterium adolescentis. FEMS Microbiol. Lett..

[47] Chia L.W., Hornung B.V.H., Aalvink S., Schaap P.J., de Vos W.M., Knol J., Belzer C. (2018). Deciphering the trophic interaction between Akkermansia muciniphila and the butyrogenic gut commensal Anaerostipes caccae using a metatranscriptomic approach. Antonie van Leeuwenhoek.

[48] Łagowska K., Drzymała-Czyż S. (2022). A low glycemic index, energy-restricted diet but not Lactobacillus rhamnosus supplementation changes fecal short-chain fatty acid and serum lipid concentrations in women with overweight or obesity and polycystic ovary syndrome. Eur. Rev. Med. Pharmacol. Sci..

