# Supplementary material for: A Pilot Study: The Reduction in Fecal Acetate in Obese Patients after Probiotic Administration and Percutaneous Electrical Neurostimulation
Source: Nutrients. 2023 Feb 21;15(5):1067. doi: 10.3390/nu15051067 (PMC10005340; doi:10.3390/nu15051067)
Supplement: Supplementary file 1 [file nutrients-15-01067-s001.zip › nutrients-2228448-supplementary.pdf]

A

Time (min)	H2O + 0.01% FA	ACN + 0.01% FA
0	80%	20%
2	80%	20%
7	60%	40%
7.5	0%	100%
8	0%	100%
11	80%	20%

B

Metabolite	Q1 (m/z)	Q3 (m/z)	Collision energy (V)
Acetate	194.0	152.10/137.05	18/21
Propionate	208.2	137.05/165.15	20/15
Butirate	222.1	137.00/152.05	20/16

Supplementary Table S1.
